# Validation of the Portuguese Version of the Quality of Life Impact of Refractive Correction Questionnaire (QIRC): Study on a Sample of Young Adults

**DOI:** 10.1590/1980-549720260047

**Published:** 2026-08-28

**Authors:** Catarina Costa Balseiro, Mariana Costa Cunha, Marco Miguel, António João Santos Nunes, Amélia Fernandes Nunes

**Affiliations:** I Universidade da Beira Interior – Covilhã, Portugal.; II Shamir Optical Portugal – Vilar, Portugal.; III Universidade da Beira Interior, Research Center for Business Sciences – Covilhã, Portugal.; IV Universidade da Beira Interior, Research Center for Health Sciences – Covilhã, Portugal.

**Keywords:** Quality of life, Visual correction, Validation studies, Questionnaires, Psychometrics.

## Abstract

**Objetive:**

In this study, we aimed to perform the cross-cultural adaptation and psychometric evaluation of the Quality of Life Impact of Refractive Correction (QIRC) questionnaire for European Portuguese.

**Methods:**

International guidelines for the adaptation of questionnaire-based assessment instruments were followed, and the study was conducted in two phases: 1) Cross-cultural adaptation, involving translation, back-translation, and pretesting for semantic equivalence; 2) Psychometric evaluation of the scale. A convenience sample was selected from secondary and higher education institutions. Reliability was assessed using Cronbach’s alpha (α) and McDonald’s omega (ω), while temporal stability was evaluated by the intraclass correlation coefficient (ICC). The evaluation of the instrument’s dimensional structure was performed using exploratory factor analysis (EFA).

**Results:**

The sample consisted of 373 students, aged between 16 and 35 years. Internal consistency was high (α=0.848 and ω=0.862) and temporal stability was considered excellent (ICC=0.951). We identified three factors (Difficulties and Concerns – 11 items; Comfort and Well-being – 7 items; and Function and Symptoms – 2 items), according to the EFA, which explained 66.82% of the total variance. Internal consistency was excellent for Difficulties and Concerns (α=0.970), good for Comfort and Well-being (α=0.830); and low for Function and Symptoms (α=0.253), the latter being composed of only two items.

**Conclusion:**

The results support the evaluation of the instrument’s dimensional structure and reliability of the Portuguese version of the QIRC, adapted for European Portuguese. It is a suitable tool for assessing the impact of refractive correction on the quality of life among young populations in Portugal.

## INTRODUCTION

Vision health is fundamental to quality of life. Its importance is such that, when compromised, it affects several dimensions of the life cycle, including development and learning capacity (in children), social relations, emotional well-being, independence, safety, and productivity at work^1^.

According to the World Health Organization (WHO), over 2.2 million people live with vision impairments^2^. Overall, uncorrected refractive errors (myopia, hyperopia, and astigmatism) are among the most prevalent vision disorders^3^. Although these are clinically controllable conditions, their impact goes beyond reducing visual acuity. Eye fatigue and excessive visual exertion are frequent causes of headache complaints, especially in adolescents and young adults^4^.

The scientific literature shows not only the vast occurrence of vision impairments due to refractive errors, but also the lack of a consistent assessment of their effects on multiple aspects of quality of life^5,6^. Uncorrected refractive errors have been associated with low self-esteem, academic and professional difficulties, social exclusion, and lower overall well-being^1^. People with reduced visual acuity have up to three times more chances of reporting difficulties in performing everyday activities^7^. Such evidence supports the relevance of validating scales sensitive to the individual’s perception of the effects of vision-related changes on well-being and function^8^.

Several instruments have been developed with this purpose, such as the Refractive Status and Vision Profile and the NEI-RQL-42, created to evaluate the results of refractive surgery, but which have limitations in the differentiation between types of optical correction^9^. The Quality of Life Impact of Refractive Correction (QIRC) specifically assesses the impact of refractive correction on quality of life, whether with glasses, contact lenses, or surgery. Its original version, validated in the English language, demonstrated reliability, internal consistency, and sensitivity to distinguish the effects of different types of correction^10,11^. QIRC is currently translated and adapted to several languages and cultures^12,13,14,15,16^.

In this study, we aimed to perform the translation, cross-cultural adaptation, and psychometric evaluation of QIRC into European Portuguese, providing a valid and reliable tool to evaluate the impact of refractive correction on the quality of life in Portugal.

## METHODS

This study was developed in two distinct phases:

1. Translation and cross-cultural adaptation of the instrument; and
2. Psychometric evaluation of the scale.

### Participants’ selection

For the translation and linguistic adaptation of the questionnaire, pretests (n=24) and cognitive interviews (n=10) were carried out with young adults recruited in a class of students attending the first year of higher education. These participants were not included in the subsequent stages of the study.

The psychometric evaluation was performed based on the answers to the final version of the questionnaire, which was self-administered to 373 students of secondary and higher education. The participants were recruited by convenience at the end of the classes, after prior consultation with the responsible teachers. The sample included both students who usually wore some type of visual correction, comprising glasses and/or contact lens, and those who did not. From this sample, a subgroup of 70 participants completed the questionnaire about one month later to evaluate the test-retest reliability.

All participants of legal age were previously informed about the objectives of the study and agreed to voluntarily participate by signing the Informed Consent Form. Students under 18 years old presented the Informed Consent Form signed by the legal guardian. The inclusion criteria were: to be a student, age between 16 and 35 years, to understand the Portuguese language, and to accept to participate voluntarily. Foreign participants whose mother tongue was not Portuguese and with less than five years of residence in Portugal, those with a history of eye pathology, and those who returned incomplete questionnaires were excluded.

### Methods and procedures

#### Structure of the Quality of Life Impact of Refractive Correction questionnaire

The QIRC questionnaire consists of 20 items distributed into five domains: function, symptoms, convenience, economic concerns, and emotional health/well-being. Each item is evaluated on a five-point Likert scale, ranging from “none” to “extreme” or from “never” to “always” depending on the domain. In addition, it includes the “Not applicable” option, which is considered as missing data in data analysis^10^.

#### Translation and cross-cultural adaptation

This process was based on the standard procedure suggested by other authors^17,18,19^ and summarized in Table 1. For this step, a committee of seven specialists from different fields of activity was assembled: three eye care professionals with over 10 years of professional experience (two optometrists and one orthoptist), one general and family doctor, one psychologist, and two behavioral professionals (human resources and sociology). The steps that constituted this process included direct translation, back-translation, and pretest.

**Table 1:** Summary of the process of translation and adaptation of the Quality of Life Impact of Refractive Correction Questionnaire for the Portuguese language.

Step	Action	Result
Direct translation	Two independent translations	Synthesis Version
	1st meeting	
Back-translation	1st back-translation	Translated Consensual Version
	2nd meeting	
Final review	Pretest, translated version	Final Version
	3rd meeting	

Source: Prepared by the authors.

The direct translation of the questionnaire was carried out by two independent bilingual translators: one optometrist and one behavioral professional. The Synthesis Version (SV) resulted from the first consensus meeting between the two translators and a third person, also from the eye care field. In the next step, a third translator, without prior knowledge of the instrument, carried out the back-translation. The back-translation was analyzed in a second consensus meeting that included the entire committee (eye care professionals, one doctor, one psychologist, and two behavioral professionals), originating the Translated Consensual Version (TCV). This version was submitted to pretest by self-administration of the questionnaire, with the supervision of an eye care professional and a psychologist. We sought to identify difficulties in understanding and autonomy in responding to the instrument. The option “I do not understand the question” was added and comments on doubts were collected, through cognitive interview. After this phase, the QIRC questionnaire was translated into *Impacto da Correção Refrativa na Qualidade de Vida (ICR-QV)*, in European Portuguese, presented in Annex A.

#### Psychometric evaluation

The sample was obtained by convenience, comprising secondary and higher education students. The recruitment in secondary education was preceded by an authorization request, and only the students who presented the Informed Consent Form signed by the parents or guardians, and after obtaining students’ Informed Assent Form on the day of the evaluation, participated. In higher education, recruitment was performed in the classrooms, after authorization of the responsible professor, and participation was based on Informed Consent given by students themselves. This sample was independent of that used in the pretesting phase.

The psychometric evaluation of the scale involved three main steps, performed in the SPSS v.29 software:

1. Evaluation of internal consistency via Cronbach’s alpha and McDonald’s omega, for greater robustness in heterogeneous samples;
2. Analysis of temporal stability (test-retest) via intraclass correlation coefficient (ICC) and Bland-Altman*,* with interval between four and six weeks, in a subsample (n=70); and
3. Evaluation of the dimensional structure of the instrument.

Internal consistency was assessed to determine the degree of interrelation between items, i.e., to verify whether the different items of the scale consistently measure the same underlying construct (impact of refractive correction on quality of life). This analysis was evaluated by Cronbach’s alpha coefficient (α) and McDonald’s omega (ω), considering acceptable values of α or ω≥0.70^20^.

Temporal stability was evaluated by test-retest, using the ICC to estimate the reliability between the two application moments, and the paired Student’s t-test was employed to verify the existence of significant differences in the means. The agreement between the measurements was analyzed using the Bland-Altman plot. A good temporal stability was considered for ICC≥0.75 values^21^, together with the absence of statistically significant differences. For calculating the scores, the answers were previously converted to a scale from 0 to 100 based on the conversion matrix provided by the authors of the original instrument. This standardization aims to ensure the comparability of scores with previous studies, as well as a continuous metric more appropriate for statistical analysis.

When assessing the dimensional structure of the instrument, an exploratory approach was chosen, as the dimensional structure of the QIRC is not widely established by confirmatory studies and some original dimensions include only one item. Thus, the exploratory factor analysis (EFA) was used to investigate the latent structure of the Portuguese version. Data adequacy was verified by the Kaiser-Meyer-Olkin (KMO) index and Bartlett’s test of sphericity. The extraction of the factors was based on the analysis of the main components, with retention of factors with eigenvalue>1 (Kaiser’s criterion), supported by the inspection of the scree plot. In addition, a minimum factor load of 0.40 was established as the criterion for the permanence of the item, ensuring that each item significantly contributed to its factor. To facilitate the interpretation of the factors, a Varimax orthogonal rotation^22^ was applied.

The study obtained a favorable opinion from the Ethics Committee of Universidade da Beira Interior (Process No. CE-UBI-Pj-2025-006-ID7056) and was carried out at the UBImedical facilities, in vision research laboratories. All procedures followed the ethical principles of the Declaration of Helsinki.

##### Data Availability Statement:

The entire dataset that supports the results of this study is available upon request to the corresponding author. The dataset is not publicly available due to the integration of databases created by the authors.

## RESULTS

### Translation and cross-cultural adaptation

In the first stage of the process, direct translation, we recorded some difficulties in the interpretation of items 1 and items 3 to 7 of the original questionnaire, which required panel discussion to reach consensus.

In item 1, “How much difficulty” was translated into *Quanta dificuldade* and *Que dificuldade*, having opted for *Que dificuldade*, for generating a more fluid and adequate reading to the structure of the question. The term “Glare” has resulted in different translations, such as *encandeamento*, *ofuscamento*, and *deslumbramento*. The term *encandeamento* was chosen, because it is more common and understandable in the context of driving.

Between items 3 and 7, the expression “How much trouble” was recurrent, initially translated as *Quantos problemas* or *Que dificuldade*. After analysis, we decided to maintain the expression *Que dificuldade*, as it ensures greater fluidity and terminological consistency throughout the scale. In item 3, the expression “off-the-shelf (nonprescription) sunglasses” was translated as *óculos de sol prontos a usar (sem prescrição)* or *óculos de sol comuns (não graduados)*, having been adjusted to *óculos de sol comuns (sem graduação)* because it is clearer and widely understood. These decisions were made based on the principle of semantic equivalence and the adequacy to the cultural and linguistic context of European Portuguese.

The second stage of the process, back-translation, went smoothly.

In the final review, a class of 30 students was recruited, comprised of students attending the first year of higher education, of which 24 effectively participated and six did not give consent. In the results of the first 10 volunteers, through cognitive interview (41.7% of the pretest group), we identified difficulties in understanding question 1 due to the term *encandeamento*. We verified no significant gains in the cognitive interview by replacing the term with *ofuscamento* or *deslumbramento*, as the three terms were considered unusual in everyday language. The suggestion of the participants was to add an explanation, recommending the inclusion of *como em situações de alta luminosidade* (“like in situations of blinding glare”) at the end of the question. Furthermore, one participant reported difficulties in interpreting item 4, for being too extensive. The cognitive interview indicated that the statement *ao pensar nos seus óculos, nas suas lentes de contacto ou nos seus olhos após a cirurgia refrativa, antes de…* (“when thinking about your glasses, your contact lenses, or your eyes after refractive surgery, before…”) could be simplified to *ao pensar na sua correção visual, antes de…* (“when thinking about your visual correction, before…”).

After these adjustments, the final version, called *Impacto da Correção Refrativa na Qualidade de Vida (ICR-QV)*, kept the original 20 items and was fully understood by the remaining 14 volunteers (58.3% of the pretest group).

### Psychometric evaluation of the scale

#### Sample characterization

373 students, 140 (37.5%) of secondary education and 233 (62.5%) of higher education, participated in the study. The mean age was 19.8±2.9 years (ranging between 16 and 35 years old). Regarding sex, we verified a balanced distribution, with 201 (53.9%) women and 172 (46.1%) men. As for the use of visual correction devices, 187 (50.1%) wore glasses or contact lenses and 186 (49.9%) did not wear any kind of visual correction device.

#### Consistency of responses and temporal stability

The internal consistency analysis of the data was evaluated by Cronbach’s alpha coefficient and McDonald’s omega. We verified high values of alpha (α=0.885) and omega (ω=0.862), attesting to a good homogeneity of the instrument in the study sample.

The temporal stability of the instrument was evaluated with data collected at two different moments, within an interval of 4 to 6 weeks, in a smaller sample (n=70). In the total scores obtained in the first and second application, we found means of 44.96±5.9 and 44.31±6.42, respectively. The distribution of responses was deemed normal at both moments, as indicated by the Shapiro-Wilk test (p>0.05). In the comparison of the means between the two moments (paired t-test), we did not find statistically significant differences (t(69)=1.221; p=0.226), suggesting a good temporal stability. To evaluate the agreement between the two applications, the ICC was calculated, whose value was 0.848 (95% confidence interval [CI] 0.756–0.906), which indicates a high degree of agreement.

**Figure 1:**
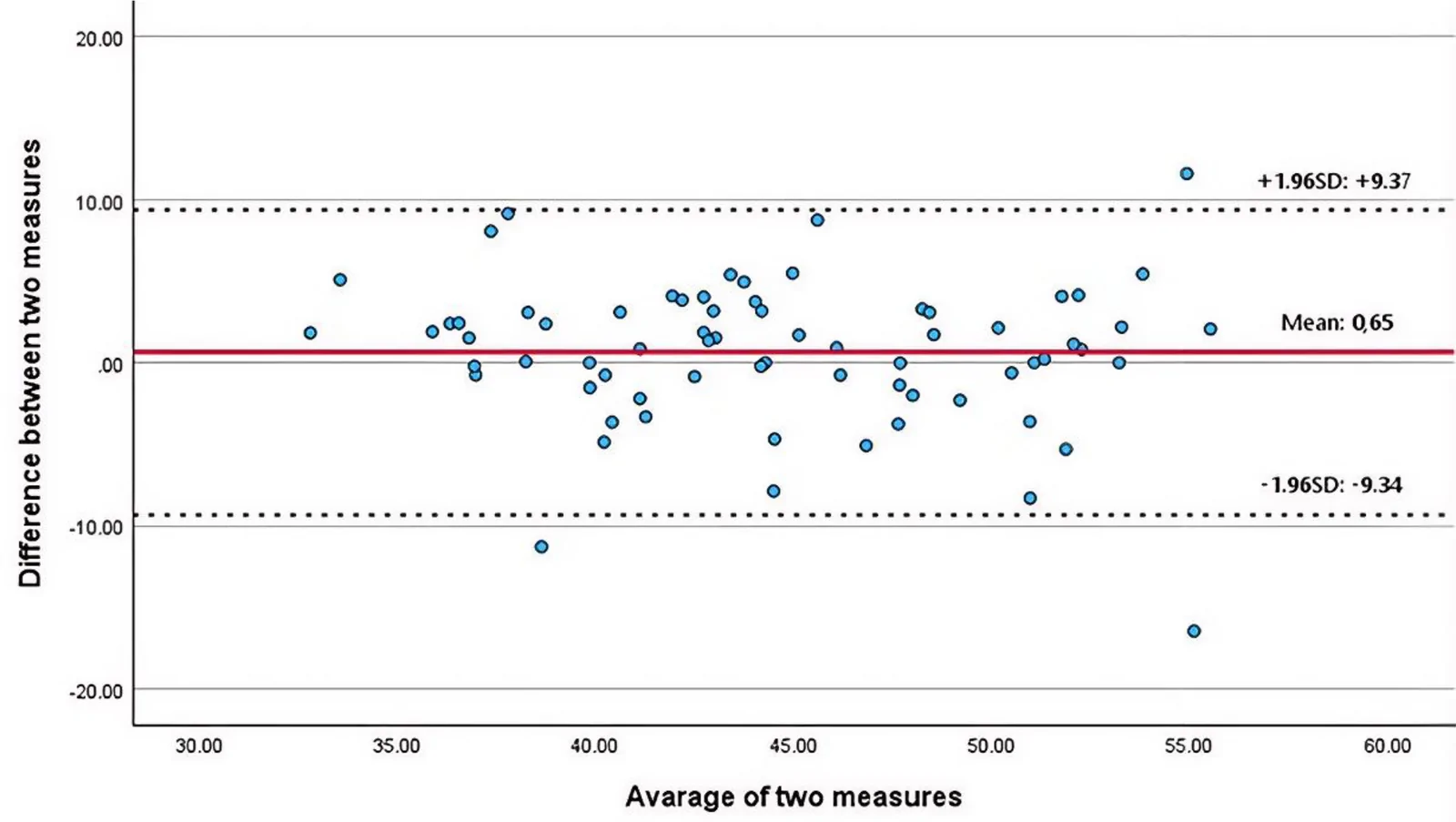
Bland-Altman plot for the study of agreement between measures.

As per the Bland Altman plot (Figure 1), the mean difference is close to zero, and the obtained variations are mostly within the 95% confidence interval. There is no proportion bias (p=0.303) according to the linear regression of the mean difference.

The center line marks the difference means. Dotted lines mark the agreement thresholds in the 95% interval.

Source: Prepared by the authors.

#### Factor structure of the instrument

**Figure 2:**
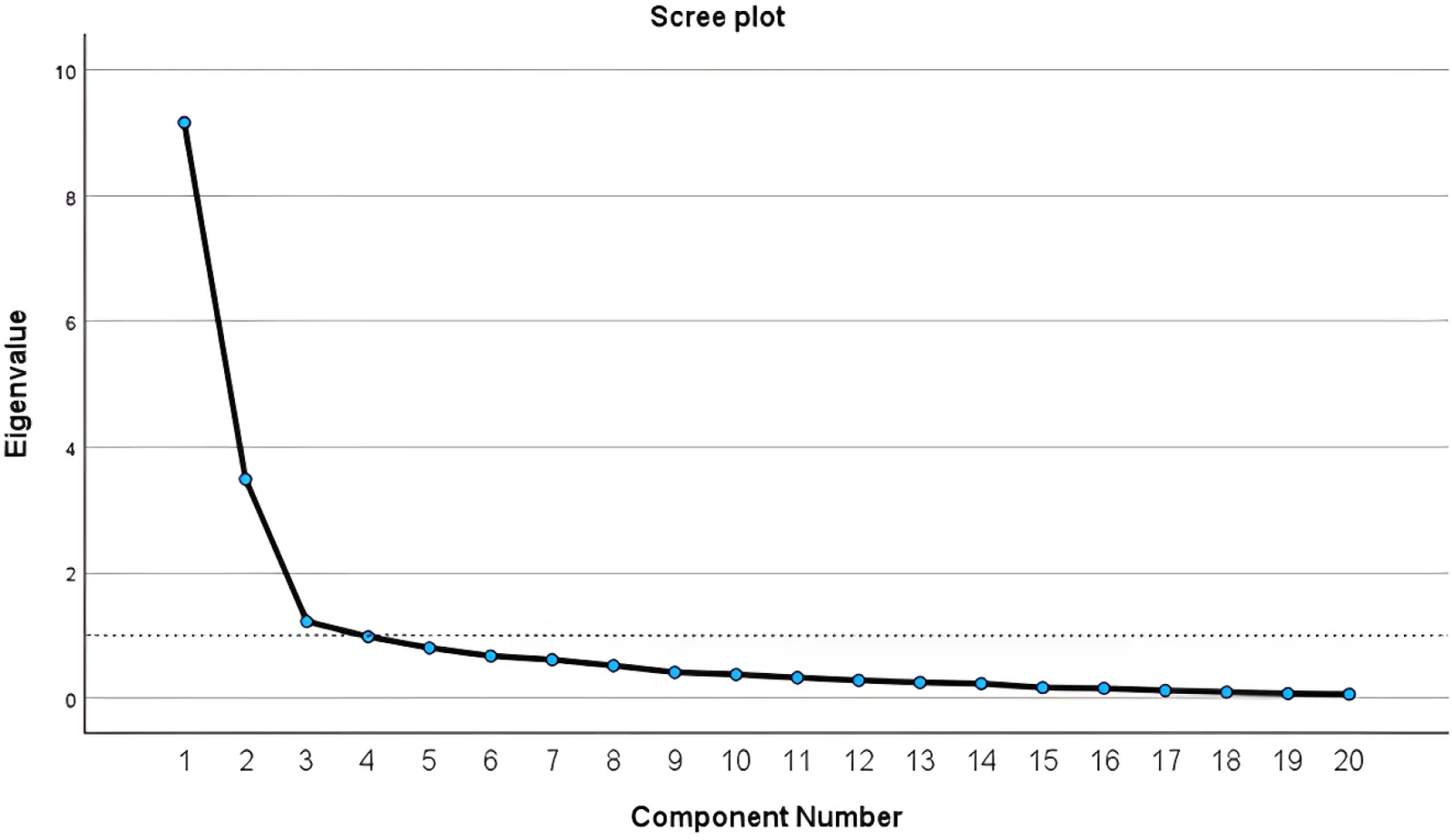
Scree plot of the exploratory factor analysis, indicating the variance explained by each component.

The latent structure of the scale was investigated by EFA (Table 2). The correlation matrix was deemed adequate for factor analysis, with an excellent value of KMO*=*0.928 and a significant Bartlett’s test of sphericity (p<0.001). According to the extraction by main components, we verified three latent factors, with eigenvalues greater than 1, according to the Kaiser-Guttman criterion and the visual inspection of the scree plot (Figure 2).

**Table 2:** Factor loads after rotation, total explained variance, denomination of the extracted factors, and internal consistency.

Item	F1	F2	F3
1. Vision when driving	0.097	-0.004	**0.542**
2. Visual symptoms	0.062	-0.198	**0.733**
3. Visual limitations with sunglasses	**0.823**	-0.028	0.022
4. Visual limitations in specific activities	**0.894**	-0.005	0.024
5. Visual limitations when waking up	**0.840**	-0.016	0.068
6. Visual limitations on the beach or pool	**0.879**	0.000	0.063
7. Visual limitations in sports practice	**0.812**	-0.015	0.102
8. Concerns about refractive material costs	**0.904**	-0.010	-0.021
9. Concerns about maintenance of visual correction	**0.895**	-0.055	-0.007
10. Concerns about dependence on refractive material	**0.936**	-0.067	0.021
11. Concerns about vision	**0.936**	-0.057	0.028
12. Concerns about medical complications	**0.882**	-0.088	0.030
13. Concerns about protection from solar radiation	**0.840**	-0.025	0.125
14. Feeling at your best	-0.059	**0.685**	0.047
15. Being seen as you wish	-0.059	**0.616**	0.401
16. Feeling praised	-0.040	**0.697**	0.405
17. Feeling confident	-0.045	**0.798**	-0.081
18. Feeling happy	-0.007	**0.790**	-0.215
19. Being able to do what you want	0.025	**0.761**	-0.169
20. Willingness to try new things	-0.029	**0.584**	-0.132
Explained variance	42.48%	17.87%	6.47%
Denomination of the extracted factors	Difficulties and concerns	Comfort and well-being	Function and symptoms
Internal consistency – Cronbach’s alpha	0.970	0.830	0.253

Values in bold indicate the highest factor load of each item.

Source: Prepared by the authors.

The inflection (“elbow”) of the plot and the Kaiser criterion (eigenvalues>1) suggest the retention of three factors.

Source: Prepared by the authors.

The extracted factor solution explained 66.82% of the total variance of the questionnaire. The first factor, called “Difficulties and Concerns,” included 11 items and explained 42.48% of the variance. The second factor, called “Comfort and well-being,” grouped 7 items related to visual comfort and emotional well-being, explaining 17.87% of the variance. The third factor, called “Function and symptoms,” included 2 items related to visual function and visual symptoms, explaining 6.47% of the variance.

Considering the internal consistency of the extracted factors, Factor 1 and Factor 2 presented α>0.70, which suggests good internal reliability. In turn, Factor 3, composed of only two items, presented a value of α=0.253, well below the threshold generally deemed acceptable. This result suggests a possible fragility in the internal cohesion of this factor, possibly related to the reduced number of items that compose it and/or its thematic heterogeneity.

## DISCUSSION

The adaptation of QIRC for European Portuguese fills an important gap in ophthalmologic evaluation in Portugal, providing a robust basis for the application of this instrument in other Portuguese-speaking countries. Although there are regional lexical specificities, the standardization of this tool in Portuguese facilitates the exchange of clinical and scientific data between Lusophone communities, enabling cross-cultural comparative studies. Traditional clinical practice often focuses on objective measures of visual acuity; however, as highlighted by the literature, these metrics do not fully capture the psychosocial and functional impact of refractive errors^23^.

For this study, the age group of the sample was defined by the criteria of the authors of the original instrument, seeking to exclude participants with presbyopia (age-related near vision loss), a condition that could compromise the validity of the answers^10^.

The adaptation of QIRC to the Portuguese language required semantic adjustments to ensure that the items were understood by different age groups. Forming a multidisciplinary committee and carrying out pretests ensured the adequate understanding of the items by the participants, allowing us to detect and correct linguistic and cultural differences. This attention to everyday language, without disrespecting the original content, is paramount to preserve the instrument’s content validity, especially in subjective domains such as quality of life^12,15^.

The psychometric evaluation showed good reliability, and the temporal stability of the scale was also confirmed, with consistent results between the two application moments. The high ICC value, the absence of significant mean differences, good agreement in the Bland-Altman plot, and the absence of systematic bias in repeated measurements indicate high temporal reliability^21^.

Our results corroborate those reported by the original author, who, considering the tool with a one-dimensional structure, presented Cronbach’s alpha coefficient values of ICC similar to our findings^10^. Moreover, our findings also corroborate those reported in other linguistic versions and demonstrate psychometric properties comparable to those validated in other languages and cultural contexts—namely, the original English questionnaire, the Malay (Malaysia), Chewa (Malawi), and Spanish (Spain) versions^12,15,16^.

The original tool postulates a one-dimensional structure; however, it is presented with different thematic domains. The exploratory factor analysis applied to our data showed a three-dimensional structure that explained a substantial proportion of the total variance. The factor structure of the tool was also reported by other authors who adapted this scale to other cultures and languages such as the Malay version^15^. For these authors, the five initial thematic domains were aggregated into only two: a functional domain and an emotional domain. In the present study, we identified three factors, one related to difficulties and concerns, another to well-being, and a third that groups function and symptoms.

Factor 1 (difficulties and concerns) grouped items 3 to 13, which in the original questionnaire belong to two distinct domains: difficulties experienced with visual correction and concerns^10,11^. This grouping in a single factor suggests that, for the young Portuguese population, the logistical challenges of visual correction and aesthetic/functional concerns are intrinsically linked, forming a unique construct. The high internal consistency of this factor, on the one hand, points to good internal cohesion of the responses and to the fact that all items measure the same construct very accurately; on the other hand, it may also indicate some redundancy between items^20^. This aspect was also noticed by other authors in different validations^15^, and even if it may indicate some redundancy, it preserves the sensitivity of the instrument to detect the impact of correction on everyday life. Factor 2 (well-being) kept the consistency with the original thematic domain of social and emotional well-being^10^, presenting good internal consistency. This consistency is also consistent with the findings of the Malay version^15^.

Factor 3, which comprises only two conceptually distinct items (visual function and visual symptoms)^10^, was grouped according to statistical criteria, and not necessarily thematic coherence. According to the literature, factors with only two items tend to have limited reliability and are particularly sensitive to low correlation between them^24^. The weak correlation between these items compromises the internal cohesion of the factor and raises doubts about its usefulness as an independent dimension. This difficulty was accentuated by the specificity of the content of Item 1 (vision when driving). The high rate of missing responses in this item reflects the demographic reality of the sample (students who do not drive yet), which compromised the cohesion of this domain. This phenomenon stresses that, although the ICR-QoL is a robust tool, its application to very young populations should consider that certain domains of quality of life depend on the respondents’ life experience and autonomy.

Despite the statistical fragility of the third factor, we chose to maintain all items in this phase of the questionnaire’s adaptation. This decision is based on the preservation of the theoretical value of the original construct and the need to ensure comparability with international studies using the same reference. Hence, we consider that the Portuguese version of the QIRC, used to evaluate the impact of refractive error on quality of life (ICR-QoL), can be safely used to assess the impact of refractive correction on the quality of life of young adults, especially in the dimensions of difficulties/concerns and emotional well-being.

This study has limitations. First, a convenience sampling was used, composed exclusively of students, which limits the generalization of the results to other age groups and sociodemographic contexts. The external validity of the instrument should be evaluated in future studies with more heterogeneous samples. Second, this research focused on cross-cultural adaptation and initial psychometric evaluation, having not explored the sensitivity of the instrument to distinguish specific groups such as people affected with myopia and hyperopia. Third, only EFA was carried out, without confirmatory factor analysis (CFA) or global indices for model adjustment. Thus, authors of future studies should confirm the dimensional structure identified in independent samples and evaluate the stability of the factor solution in different populations.

The Portuguese version of the QIRC presented satisfactory results in the initial psychometric evaluation, with good overall internal consistency and high temporal stability. As per the exploratory factor analysis, we identified a dimensional structure that differs from the original proposal, both in number and in the composition of the factors, in such a way that these findings should be carefully interpreted. Therefore, it is recommended that future studies evaluate the dimensional structure of the instrument by CFA, in independent and more heterogeneous samples. Nonetheless, the results indicate that the ICR-QoL can be a useful tool to evaluate the impact of refractive correction on quality of life in young Portuguese-speaking populations.

## Supplementary Materials

Annex A
