# Supplementary material for: Validation of the Portuguese Version of the Quality of Life Impact of Refractive Correction Questionnaire (QIRC): Study on a Sample of Young Adults
Source: Rev Bras Epidemiol. 2026 Aug 28;29:e260047. doi: 10.1590/1980-549720260047 (PMC13523783; doi:10.1590/1980-549720260047)
Supplement: Supplementary file 1 [file 1980-5497-rbepid-29-e260047-suppl1.pdf]

Anexo A

Impacto da Correção Refrativa na Qualidade de Vida - CRQV

Assinale com um "X" a condição que melhor representa a sua situação atual.

Já realizei **cirurgia refrativa** (LASIK, PRK etc.).		Vá para as questões do grupo A
Uso óculos e/ou lentes de contacto durante **todo o tempo** em que estou acordado,		Vá para as questões do grupo B
Uso óculos e/ou lentes de contacto de **forma parcial** (apenas algumas horas do dia)		Vá para as questões do grupo C
**Não uso** óculos nem lentes de contacto, e nunca fiz cirurgia refrativa		Vá para a página 3

Grupo A – Cirurgia Refrativa

A1- Há quanto tempo fez a cirurgia refrativa? __________ (anos / meses) [riscar o que não interessar]

A2- Depois da cirurgia refrativa, **ainda usa óculos ou lentes de contacto**? Não ☐ Sim ☐

A3- Se ocasionalmente ainda usa óculos e/ou lentes de contacto DESDE a sua cirurgia refrativa, por favor, estime quantas horas por dia, em média os utiliza. (Óculos de sol comuns NÃO contam como óculos de correção)

Óculos: ___________horas por dia

Lentes de contacto: ___________horas por dia **Vá para a página 3**

Grupo B – Óculos ou lentes de contacto o TEMPO TODO

B.1 - Assinale/preencha as caixas apropriadas, considerando a sua correção ótica atual. (Óculos de sol comuns não são considerados óculos de correção)

☐ Apenas óculos o tempo todo

☐ Apenas lentes de contacto o tempo todo

☐ Ambos, óculos e lentes de contacto. Óculos: ____________horas por dia

Lentes de contacto: __________ horas por dia **Vá para a página 3**

Grupo C – Óculos ou lentes de contacto a TEMPO PARCIAL

C-1 - Assinale/preencha as caixas apropriadas considerando a sua correção ótica atual. Os óculos de sol comuns NÃO contam como óculos de correção.

Apenas óculos. Usados em tempo parcial		Óculos: ____________horas por dia
Apenas lentes de contacto. Usadas em tempo parcial		Lentes de contacto: ____________horas por dia
Ambos, óculos e lentes de contacto.		Óculos: __________________ horas por dia<br>Lentes de contacto: ___________horas por dia

Vá para a página 3

ICR – QV

Por favor, preencha todas as questões relacionadas com os seus óculos ou lentes de contacto atuais. Se realizou cirurgia refrativa deve responder como está AGORA e não como estava antes da cirurgia.

Siga as instruções do exemplo inicial assinalando a caixa correta para quando usa óculos, Lentes de contacto e para quando não está a usar nenhuma correção ótica:

O: A sua resposta para quando usa **Ó**culos;

LC: A sua resposta para quando usa **L**entes de **C**ontacto;

N: A sua resposta para quando **N**ÃO usa óculos nem lentes de contacto**.**

(Se usa óculos ou LC ocasionalmente, considere o método que é usado com maior frequência)

---

1. Que dificuldade tem ao conduzir em condições de encandeamento (como em situações de alta luminosidade)?

Não aplicável	Não conduzo por outras razões (não visuais)	Nenhuma	Pouca	Moderada	Muita	Tanta que não posso conduzir

2. Durante o último mês, com que frequência sentiu os seus olhos cansados ou tensos?

Não aplicável	Nunca	Ocasionalmente	Às vezes	Muitas Vezes	Sempre

	Não aplicável	Nenhuma	Pouca	Moderada	Bastante	Extrema
3. Que dificuldade sente por não poder usar óculos de sol comuns (sem graduação)?						
4. Que dificuldade sente ao pensar na sua correção visual, antes de fazer certas atividades; Como por exemplo: viajar, praticar desporto, nadar?						
5.Que dificuldade sente quando acorda e não consegue ver bem, como por exemplo, para ir à casa de banho, cuidar de um bebé ou ver o despertador?						
6.Que dificuldade sente ao não conseguir ver bem na praia ou ao nadar no mar/piscina, porque faz estas atividades sem óculos ou sem lentes de contacto?						
7.Que dificuldade sente ao usar óculos ou lentes de contacto quando vai ao ginásio, nas aulas de ginástica, no treino em circuito, etc.?						

	Não aplicável	Nenhuma	Pouca	Moderada	Bastante	Extrema
8.Que preocupação tem com o custo inicial e contínuo de compra dos seus óculos, lentes de contacto ou cirurgia refrativa?						
9.Que preocupação tem com o custo da manutenção não programada dos seus óculos, lentes de contacto ou cirurgia refrativa, tais como quebras, perdas ou novos problemas oculares?						
10. Que preocupação tem com o facto de ter que depender cada vez mais dos seus óculos ou lentes de contacto desde que começou a usá-los?						
11.Que preocupação tem sobre o facto da sua visão não ser tão boa como poderia ser?						
12.Que preocupação tem com as complicações médicas da sua escolha de correção ótica (óculos, lentes de contacto e/ou cirurgia refrativa)?						
13.Que preocupação tem com a proteção dos olhos contra a radiação ultravioleta (UV)?						

Estamos agora interessados no efeito que a sua correção ótica teve na forma como se tem sentido (óculos, lentes de contacto ou cirurgia refrativa). Esse efeito pode ser óbvio (por exemplo, sentir que fica com melhor aparência com os seus novos óculos) ou indireto (por exemplo, sentir-se mais confiante desde que começou a usar lentes de contacto ou realizou uma cirurgia refrativa, porque sente que tem melhor aparência).

	Não aplicável	Nunca	Ocasionalmente	Às vezes	Muitas Vezes	Sempre
14.Durante o último mês, com que frequência sentiu que estava no seu melhor?						
15.Durante o último mês, com que frequência sentiu que os outros o veem da maneira que gostaria que o vissem (por exemplo, inteligente, sofisticado, bem-sucedido, fixe, etc.)						
16.Durante o ultimo mês, com que frequência se sentiu elogiado/lisonjeado?						
17-Durante o último mês, com que frequência se sentiu confiante?						
18.Durante o último mês, com que frequência se sentiu feliz?						
19.Durante o último mês, com que frequência se sentiu capaz de fazer aquilo que queria fazer?						
20.Durante o último mês, com que frequência sentiu vontade de experimentar coisas novas?						
