# Supplementary material for: Validation of the Portuguese Version of the Quality of Life Impact of Refractive Correction Questionnaire (QIRC): Study on a Sample of Young Adults
Source: Rev Bras Epidemiol. 2026 Aug 28;29:e260047. doi: 10.1590/1980-549720260047 (PMC13523783; doi:10.1590/1980-549720260047)
Supplement: Translation

# Translation: Validação da versão portuguesa do questionário Impacto da Correção Refrativa na Qualidade de Vida (ICR-QV) em uma amostra de adultos jovens

**DOI:** 10.1590/1980-549720260047.2

**Authors:** Catarina Costa Balseiro, Mariana Costa Cunha, Marco Miguel, António João Santos Nunes, Amélia Fernandes Nunes

**Affiliations:** I Universidade da Beira Interior – Covilhã, Portugal.; II Shamir Optical Portugal – Vilar, Portugal.; III Universidade da Beira Interior, Research Center for Business Sciences – Covilhã, Portugal.; IV Universidade da Beira Interior, Research Center for Health Sciences – Covilhã, Portugal.

**Keywords:** Qualidade de vida, Correção visual, Estudos de validação, Questionários, Psicometria.

## Abstract

**Objetivo:**

Este estudo teve como objetivo realizar a adaptação transcultural e a avaliação psicométrica do questionário *Quality of Life Impact of Refractive Correction* (QIRC) para o português europeu.

**Métodos:**

Seguiram-se as orientações internacionais para a adaptação de instrumentos de avaliação por questionário, em duas fases: 1) Adaptação transcultural, envolvendo tradução, retrotradução e pré-teste para equivalência semântica; 2) Avaliação psicométrica da escala. A amostra foi selecionada por conveniência em instituições de ensino secundário e universitário. A confiabilidade foi avaliada pelo *alfa de Cronbach* (α) e pelo *ômega de McDonald* (ω), e a estabilidade temporal pelo Coeficiente de Correlação Intraclasse (CCI). A avaliação da estrutura dimensional do instrumento foi analisada pela Análise Fatorial Exploratória (AFE).

**Resultados:**

Participaram 373 estudantes com idades compreendidas entre 16 e 35 anos. A consistência interna global foi elevada (α=0,848 e ω=0,862) e a estabilidade temporal foi considerada excelente (CCI=0,951). A AFE identificou três fatores (Dificuldades e preocupações – 11 itens; Conforto e bem-estar – 7 itens; e Função e sintomas – 2 itens) que explicaram 66,82% da variância total. A consistência interna foi excelente para Dificuldades e preocupações (α=0,970); boa para Conforto e bem-estar (α=0,830); e baixa para Função e sintomas (α=0,253), fator composto por apenas dois itens.

**Conclusão:**

Os resultados obtidos apoiam a avaliação da estrutura dimensional do instrumento e a confiabilidade da versão portuguesa do QIRC, adaptada para o português europeu, que se apresenta como uma ferramenta adequada para avaliar o impacto da correção refrativa na qualidade de vida em populações jovens em Portugal.

## INTRODUÇÃO

A saúde visual é fundamental para a qualidade de vida, e a sua importância é tal que, quando comprometida, afeta diversas dimensões do ciclo vital, incluindo o desenvolvimento e a capacidade de aprendizagem (em crianças), as relações sociais, o bem-estar emocional, a independência, a segurança e a produtividade no trabalho^1^.

Segundo a Organização Mundial da Saúde (OMS), mais de 2,2 milhões de pessoas vivem com deficiência visual^2^. Globalmente, os erros refrativos não corrigidos (miopia, hipermetropia e astigmatismo) estão entre os problemas visuais mais prevalentes^3^. Embora sejam condições clinicamente controláveis, o seu impacto vai além da redução da acuidade visual. A fadiga ocular e o esforço visual excessivo que geram são causas frequentes de queixas de cefaleias, especialmente em adolescentes e jovens adultos^4^.

A literatura científica evidencia não só a vasta ocorrência de déficits visuais devido a erros de refrativos mas também a falta de uma avaliação consistente dos seus efeitos em múltiplos aspetos da qualidade de vida^5,6^. Os erros refrativos não corrigidos têm sido associados a baixa autoestima, dificuldades acadêmicas e profissionais, exclusão social e menor bem-estar em geral^1^. Pessoas com menor acuidade visual têm até três vezes mais chances de relatar dificuldades na execução de atividades cotidianas^7^. Tais evidências sustentam a relevância da validação de escalas sensíveis à percepção do indivíduo sobre os efeitos das alterações visuais no bem-estar e na funcionalidade^8^.

Diversos instrumentos têm sido desenvolvidos com esse objetivo, como o *Refractive Status and Vision Profile* e o NEI-RQL-42, concebidos para avaliar os resultados da cirurgia refrativa, mas que apresentam limitações na diferenciação entre tipos de correção ótica^9^. O *Quality of Life Impact of Refractive Correction* (QIRC) avalia especificamente o impacto da correção refrativa na qualidade de vida, seja com óculos, lentes de contacto ou cirurgia. A sua versão original, validada em língua inglesa, demonstrou confiabilidade, consistência interna e sensibilidade para distinguir os efeitos das diferentes formas de correção^10,11^. O QIRC, atualmente, encontra-se já traduzido e adaptado para diversas línguas e culturas^12,13,14,15,16^.

Este estudo teve como objetivo a tradução, adaptação transcultural e avaliação psicométrica do QIRC para a língua portuguesa, disponibilizando uma ferramenta válida e fiável para avaliar o impacto da correção refrativa na qualidade de vida em Portugal.

## MÉTODOS

Este estudo desenvolveu-se em duas fases distintas:

1. Tradução e adaptação transcultural do instrumento; e
2. Avaliação psicométrica da escala.

### Seleção de participantes

Para a fase de tradução e adaptação linguística do questionário, foram realizados pré-testes (n=24) e entrevistas cognitivas (n=10) com adultos jovens recrutados em uma turma do primeiro ano do ensino superior. Esses participantes não foram incluídos nas etapas subsequentes do estudo.

A avaliação psicométrica foi realizada com base nas respostas à versão final do questionário, autoaplicado a 373 estudantes do ensino secundário e universitário. Os participantes foram recrutados por conveniência, ao final das aulas, após consulta prévia aos professores responsáveis. A amostra incluiu estudantes com e sem uso habitual de correção visual, abrangendo usuários de óculos e/ou de lentes de contato. Dessa amostra, um subgrupo de 70 participantes voltou a preencher o questionário cerca de um mês depois, para avaliação da confiabilidade teste-reteste.

Todos os participantes maiores foram previamente informados sobre os objetivos do estudo e aceitaram participar voluntariamente, assinando o termo de consentimento. Os alunos com menos de 18 anos apresentaram consentimento assinado pelo tutor legal. Os critérios de inclusão foram: ser estudante, ter entre 16 e 35 anos, compreender a língua portuguesa e aceitar participar de forma voluntária. Foram excluídos os participantes estrangeiros cuja língua materna não fosse o português e com menos de 5 anos de residência em Portugal, aqueles com histórico de patologia ocular e os que devolveram questionários incompletos.

### Métodos e procedimentos

#### Estrutura do questionário Quality of Life Impact of Refractive Correction

O questionário QIRC é composto por 20 itens distribuídos por cinco domínios: função, sintomas, conveniência, preocupações econômicas e saúde/bem-estar emocional. Cada item é avaliado em uma escala de *Likert* de 5 pontos, variando entre “nenhuma” e “extrema” ou entre “nunca” e “sempre”, consoante o domínio. Adicionalmente, inclui a opção “Não se aplica”, resposta que é considerada como dado ausente na análise de dados^10^.

#### Tradução e adaptação transcultural

Este processo baseou-se no procedimento standard sugerido por outros autores^17,18,19^ e resumido na Tabela 1. Para essa etapa, foi reunido um comitê constituído por 7 especialistas de diferentes áreas de atuação: 3 profissionais de saúde ocular com mais de 10 anos de experiencia profissional (dois optometristas e um ortoptista), um médico de saúde geral e familiar, um psicólogo e dois profissionais da área comportamental (recursos humanos e sociologia). As etapas que constituíram esse processo incluíram a tradução direta, a tradução reversa e o pré-teste.

**Tabela 1:** Resumo do processo de tradução e adaptação do questionário Quality of Life Impact of Refractive Correction Questionnaire para a língua portuguesa.

Etapa	Ação	Resultado
Tradução direta	Duas traduções independentes	Versão síntese
	1^a^ reunião	
Tradução reversa	1 tradução reversa	Versão consensual traduzida
	2^a^ reunião	
Revisão final	Pré-teste, versão traduzida	Versão final
	3^a^ reunião	

Fonte: Elaborado pelos autores.

A tradução direta do questionário foi realizada por dois tradutores bilingues independentes: um profissional de saúde ocular e outro da área comportamental. A Versão Síntese (VS) resultou da primeira reunião de consenso entre os dois tradutores e um terceiro elemento, também da área da saúde ocular. Na etapa seguinte, um terceiro tradutor, sem conhecimento prévio do instrumento, realizou a tradução inversa. Essa foi analisada em uma segunda reunião de consenso que incluiu todo o comitê (profissionais de saúde ocular, um médico e um psicólogo), originando a Versão Consensual Traduzida (VcT). Essa versão foi submetida a pré-teste por autopreenchimento do questionário, com supervisão de um profissional de saúde visual e um psicólogo. Procurou-se identificar dificuldades de compreensão e autonomia na resposta. Foi adicionada a opção “não entendo a questão” e recolhidos comentários sobre as dúvidas suscitadas, mediante entrevista cognitiva. Após essa fase o questionário QIRC foi traduzido para “Impacto da Correção Refrativa na Qualidade de Vida” (ICR-QV), apresentado no anexo A.

#### Avaliação psicométrica

A amostra foi obtida por conveniência, integrando estudantes do ensino secundário e do ensino superior. O recrutamento no ensino secundário foi precedido pelo pedido de autorização, tendo apenas participado os alunos que apresentaram o Consentimento assinado pelos encarregados de educação e com obtenção de Assentimento do aluno no dia da avaliação. No ensino superior, o recrutamento foi em sala de aula, após autorização do professor responsável, e a participação baseou-se no consentimento livre dos próprios estudantes. Essa amostra foi independente da utilizada na fase de pré-teste.

O processo de avaliação psicométrica da escala envolveu três etapas principais, executadas no *software* SPSS v.29:

1. Avaliação da consistência interna via *alfa de Cronbach* e *ômega de McDonald*, para maior robustez em amostras heterogêneas;
2. Análise da estabilidade temporal (teste-reteste) via Coeficiente de Correlação Intraclasse (CCI) e *Bland-Altman*, com intervalo entre quatro e seis semanas, em uma subamostra (n=70); e
3. Avaliação da estrutura dimensional do instrumento.

A consistência interna foi avaliada para determinar o grau de inter-relação entre os itens, ou seja, para verificar se os diferentes itens da escala medem consistentemente o mesmo construto subjacente (impacto da correção refrativa na qualidade de vida). Essa análise foi avaliada pelo coeficiente *alfa de Cronbach* (α) e *ômega de McDonald* (ω), considerando-se aceitáveis valores de α ou ω≥0,70^20^.

A estabilidade temporal foi avaliada pelo teste-reteste, utilizando-se o CCI para estimar a confiabilidade entre os dois momentos de aplicação, e o teste *t de Student* emparelhado, para verificar a existência de diferenças significativas nas médias. A concordância entre as medições foi analisada por meio do gráfico de *Bland-Altman*. Considerou-se boa estabilidade temporal para valores de CCI≥0,75, em conjunto com a ausência de diferenças estatisticamente significativas^21^. Para o cálculo das pontuações, as respostas foram previamente convertidas para uma escala de 0 a 100 com base na matriz de conversão fornecida pelos autores do instrumento original. Essa padronização visa garantir a comparabilidade dos escores com estudos anteriores, bem como uma métrica contínua mais apropriada à análise estatística.

Na avaliação da estrutura dimensional do instrumento, optou-se por uma abordagem exploratória, uma vez que a estrutura dimensional do QIRC não está amplamente estabelecida por estudos confirmatórios e algumas dimensões originais incluem apenas um item. Assim, a Análise Fatorial Exploratória (AFE) foi utilizada para explorar a estrutura latente da versão portuguesa. A adequação dos dados foi verificada por meio do índice Kaiser-Meyer-Olkin (KMO) e do teste de esfericidade de Bartlett. A extração dos fatores baseou-se na análise dos componentes principais, com retenção de fatores com *eigenvalue*>1 (critério de *Kaiser*), apoiada pela inspeção do *scree plot*. Adicionalmente, estabeleceu-se como critério de permanência do item uma carga fatorial mínima de 0,40, garantindo que cada item contribuísse significativamente para seu fator. Para facilitar a interpretação dos fatores, foi aplicada rotação ortogonal do tipo *Varimax* ^22^.

O estudo obteve parecer favorável da Comissão de Ética da Universidade da Beira Interior (processo n^o^ CE-UBI-Pj-2025-006-ID7056) e foi realizado nas instalações do UBImedical, nos laboratórios de rastreio visual. Todos os procedimentos seguiram os princípios éticos da Declaração de Helsínquia.

#### Declaração de disponibilidade de dados:

Todo o conjunto de dados que dá suporte aos resultados deste estudo está disponível mediante solicitação ao autor correspondente. O conjunto de dados não está publicamente disponível devido à integração de bancos de dados realizados pelos autores.

## RESULTADOS

### Tradução e adaptação transcultural

Na primeira etapa do processo, tradução direta, registaram-se algumas dificuldades na interpretação dos itens 1 e 3 a 7 do questionário original, o que exigiu discussão em painel, para obter consenso.

No item 1, o termo “*How much difficulty*” foi traduzido para “Quanta dificuldade” e para “Que dificuldade”, tendo-se optado por “Que dificuldade”, por gerar uma leitura mais natural e adequada à estrutura da pergunta. O termo “*Glare*” gerou diferentes traduções, como “encandeamento”, “ofuscamento” e “deslumbramento”. Escolheu-se o termo “encandeamento”, por ser mais comum e compreensível no contexto da direção.

Entre os itens 3 e 7, foi recorrente a expressão “*How much trouble*”, inicialmente traduzida como “Quantos problemas” ou “Que dificuldade”. Após análise, decidiu-se manter a expressão “Que dificuldade”, por garantir maior fluidez e consistência terminológica ao longo da escala. No item 3, a expressão “*off-the-shelf* (*non prescription*) *sunglasses*” foi alvo de versões como “óculos de sol prontos a usar (sem prescrição)” ou “óculos de sol comuns (não graduados)”, tendo-se ajustado para a forma, “óculos de sol comuns (sem graduação)”, por ser mais clara e amplamente compreendida. Essas decisões foram tomadas com base no princípio da equivalência semântica e na adequação ao contexto cultural e linguístico do português europeu.

A segunda etapa do processo, tradução inversa, decorreu sem dificuldades.

Na revisão final, foi recrutada uma turma de 30 alunos, estudantes do 1^o^ ano do ensino superior, dos quais 24 participaram efetivamente e 6 não deram consentimento. Nos resultados dos primeiros 10 voluntários, com entrevista cognitiva (41,7% do grupo pré-teste), identificaram-se dificuldades na compreensão da questão 1, devido ao termo “encandeamento”. A entrevista cognitiva não revelou ganhos significativos com a substituição por “ofuscamento” ou “deslumbramento”, uma vez que os três termos foram considerados pouco usuais na linguagem do dia a dia. A sugestão dos participantes foi adicionar uma explicação, propondo a inclusão “como em situações de alta luminosidade” no final da questão. Adicionalmente, um participante referiu dificuldades na interpretação do item 4, por ser demasiado extenso. A entrevista cognitiva indicou que a formulação “ao pensar nos seus óculos, nas suas lentes de contacto ou nos seus olhos após a cirurgia refrativa, antes de…” poderia ser simplificada para “ao pensar na sua correção visual, antes de…”.

Após esses ajustes, a versão final, designada Impacto da Correção Refrativa na Qualidade de Vida (ICR-QV), manteve os 20 itens originais e foi compreendida integralmente pelos 14 voluntários restantes (58,3% do grupo pré-teste).

### Avaliação psicométrica da escala

#### Caracterização da amostra

Participaram 373 estudantes, 140 (37,5%) do ensino secundário e 233 (62,5%) do ensino superior. A média de idades foi de 19,8±2,9 anos (amplitude entre 16 e 35 anos). Relativamente ao gênero, verificou-se uma distribuição equilibrada, com 201 (53,9%) do sexo feminino e 172 (46,1%) do sexo masculino. Quanto ao uso de correção visual, 187 (50,1%) utilizavam óculos ou lentes de contacto e 186 (49,9%) não utilizavam qualquer tipo de correção.

#### Consistência das respostas e estabilidade temporal

A análise da consistência interna dos dados foi avaliada pelo coeficiente *alfa de Cronbach* e *ômega de McDonald*. Essa análise mostrou um valor elevado de *alfa* (α=0,885) e de *ômega* (ω=0,862), atestando uma boa homogeneidade do instrumento na amostra em estudo.

A estabilidade temporal do instrumento foi avaliada com coleta de dados em dois momentos distintos, num intervalo de 4 a 6 semanas, em uma amostra mais reduzida (n=70). As pontuações totais obtidas na primeira e segunda aplicação revelaram médias de 44,96±5,9 e 44,31±6,42, respetivamente. A distribuição das respostas foi considerada normal em ambos os momentos, conforme indicado pelo teste de *Shapiro-Wilk* (p>0,05). A comparação das médias entre os dois momentos (teste-*t* emparelhado) não evidenciou diferenças estatisticamente significativas (t(69)=1,221; p=0,226), sugerindo uma boa estabilidade temporal. Para avaliar a concordância entre as duas aplicações, foi calculado o CCI, cujo valor foi de 0,848 (IC95% 0,756–0,906), o que indica um grau de concordância elevado.

O gráfico de *Bland Altman* (Figura 1) mostra que a média das diferenças é próxima de zero, e as variações obtidas encontram-se maioritariamente dentro do intervalo de confiança de 95%. A regressão linear da diferença das médias mostra que não existe viés de proporção (p=0,303).

**Figura 1:** Gráfico de *Bland-Altman* para estudo da concordância entre medidas.

A linha central marca a média das diferenças. As linhas ponteadas marcam os limites de concordância no intervalo de 95%.

Fonte: Elaborado pelos autores.

#### Estrutura fatorial do instrumento

A estrutura latente da escala foi explorada por AFE (Tabela 2). A matriz de correlações foi considerada adequada à análise fatorial, com um valor excelente de *KMO*=0,928 e teste de Esfericidade de *Bartlett* significativo (p<0,001). A extração por componentes principais revelou três fatores latentes, com autovalores superiores a 1, conforme o critério de *Kaiser-Guttman* e a inspeção visual do *scree plot* (Figura 2).

**Tabela 2:** Cargas fatoriais após rotação, variância total explicada, denominação dos fatores extraídos e consistência interna.

Item	F1	F2	F3
1. Visão ao dirigir	0,097	-0,004	**0,542**
2. Sintomas visuais	0,062	-0,198	**0,733**
3. Limitações visuais com óculos de sol	**0,823**	-0,028	0,022
4. Limitações visuais em atividades específicas	**0,894**	-0,005	0,024
5. Limitações visuais ao acordar	**0,840**	-0,016	0,068
6. Limitações visuais na praia ou piscina	**0,879**	0,000	0,063
7. Limitações visuais na prática esportiva	**0,812**	-0,015	0,102
8. Preocupações com custos do material refrativo	**0,904**	-0,010	-0,021
9. Preocupações com manutenção da correção visual	**0,895**	-0,055	-0,007
10. Preocupações com a dependência do material refrativo	**0,936**	-0,067	0,021
11. Preocupações com a visão	**0,936**	-0,057	0,028
12. Preocupações com complicações médicas	**0,882**	-0,088	0,030
13. Preocupação com a proteção à radiação solar	**0,840**	-0,025	0,125
14. Sentir-se no seu melhor	-0,059	**0,685**	0,047
15. Ser visto como deseja	-0,059	**0,616**	0,401
16. Sentir-se elogiado	-0,040	**0,697**	0,405
17. Sentir-se confiante	-0,045	**0,798**	-0,081
18. Sentir-se feliz	-0,007	**0,790**	-0,215
19. Capaz de fazer o que quer	0,025	**0,761**	-0,169
20. Vontade de experimentar coisas novas	-0,029	**0,584**	-0,132
Variância explicada	42,48%	17,87%	6,47%
Denominação dos fatores extraídos	Dificuldades e preocupações	Conforto e bem-estar	Função e sintomas
Consistência interna – alfa de Cronbach	0,970	0,830	0,253

Valores em negrito indicam a maior carga fatorial de cada item.

Fonte: Elaborado pelos autores.

**Figura 2:** *Scree plot* da Análise Fatorial Exploratória, indicando a variância explicada por cada componente.

A linha central marca a média das diferenças. As linhas ponteadas marcam os limites de concordância no intervalo de 95%.

Fonte: Elaborado pelos autores.

A solução fatorial extraída explicou 66,82% da variância total do questionário. O primeiro fator, denominado “Dificuldades e preocupações”, incluiu 11 itens e explicou 42,48% da variância. O segundo fator, denominado “Conforto e bem-estar”, agrupou 7 itens relacionados ao conforto visual e ao bem-estar emocional, explicando 17,87% da variância. O terceiro fator, denominado “Função e sintomas”, incluiu 2 itens relacionados à função visual e aos sintomas visuais, explicando 6,47% da variância.

A consistência interna dos fatores extraídos revelou que o Fator 1 e o Fator 2 apresentaram α>0,70, o que sugere boa confiabilidade interna. Já o Fator 3, composto por apenas dois itens, apresentou um valor de α=0,253, muito abaixo do limiar geralmente considerado aceitável. Esse resultado sugere uma possível fragilidade na coesão interna desse Fator, possivelmente relacionada com o número reduzido de itens que o compõem e/ou com a sua heterogeneidade temática.

## DISCUSSÃO

A adaptação do QIRC para o português europeu preenche uma lacuna importante na avaliação oftalmológica em Portugal, oferecendo uma base robusta para a aplicação desse instrumento noutros países de língua oficial portuguesa. Embora existam especificidades lexicais regionais, a padronização dessa ferramenta em português facilita o intercâmbio de dados clínicos e científicos entre comunidades lusófonas, permitindo estudos comparativos transculturais. A prática clínica tradicional foca frequentemente medidas objetivas de acuidade visual; contudo, como sublinhado pela literatura, essas métricas não captam totalmente o impacto psicossocial e funcional dos erros refrativos^23^.

Para esse estudo, a faixa etária da amostra foi definida pelos critérios dos autores do instrumento original, de forma a excluir participantes com presbiopia, condição que poderia comprometer a validade das respostas^10^.

A adaptação do QIRC para a língua portuguesa exigiu ajustes semânticos para garantir que os itens fossem compreendidos por diferentes faixas etárias. A constituição de um comitê multidisciplinar e a realização de pré-testes asseguraram a compreensão adequada dos itens por parte dos participantes, permitindo detectar e corrigir diferenças linguísticas e culturais. Essa atenção à linguagem cotidiana, sem desrespeitar o conteúdo original, é fundamental para preservar a validade de conteúdo do instrumento, especialmente em domínios subjetivos como a qualidade de vida^12,15^.

A avaliação psicométrica demonstrou boa confiabilidade, e a estabilidade temporal da escala foi igualmente confirmada, com resultados consistentes entre os dois momentos de aplicação. O alto valor do CCI, a ausência de diferenças significativas nas médias, a boa concordância no gráfico de *Bland-Altman* e a ausência de um viés sistemático nas medições repetidas indicam elevada confiabilidade temporal^21^.

Os nossos resultados estão em concordância com os reportados pelo autor original, que, considerando a ferramenta com uma estrutura unidimensional, apresentou valores de coeficiente de *alfa de Cronbach* de ICC semelhantes aos dos nossos resultados^10^. Além disso, os nossos achados também vão ao encontro do reportado noutras versões linguísticas e demonstra propriedades psicométricas comparáveis às versões validadas noutras línguas e em contextos culturais — nomeadamente, o questionário inglês original, o malaico (Malásia), o chichewa (Malawi) e o espanhol (Espanha)^12,15,16^.

A ferramenta original postula uma estrutura unidimensional; contudo, ela é apresentada com diferentes domínios temáticos. A análise fatorial exploratória aplicada aos nossos dados revelou uma estrutura tridimensional que explicou uma proporção substancial da variância total. A estrutura fatorizável da ferramenta foi também reportada por outros autores que efetuaram adaptação dessa escala para outra cultura e língua, como é o caso da versão malaica^15^. Para esses autores, os cinco domínios temáticos iniciais foram agregados em apenas dois: um domínio funcional e um domínio emocional. No presente estudo, foram identificados três fatores, um relativo a dificuldades e preocupações, outro sobre o bem-estar e um terceiro que agrupa itens de função e sintomas.

O fator 1 (dificuldades e preocupações) agrupou os itens 3 a 13, que no questionário original pertencem a dois domínios distintos: dificuldades sentidas com a correção visual e preocupações^10,11^. Esse agrupamento em um único fator sugere que, para a população portuguesa jovem, os desafios logísticos da correção visual e as preocupações estéticas/funcionais estão intrinsecamente ligados, formando um construto único. A alta consistência interna desse fator, por um lado, aponta para uma boa coesão interna das respostas e para que todos os itens medem o mesmo construto com muita precisão, e, por outro lado, também pode indicar alguma redundância entre itens^20^. Esse aspeto foi também notado por outros autores noutras validações^15^, e mesmo podendo indicar alguma redundância, preserva a sensibilidade do instrumento para detectar o impacto da correção no cotidiano. O fator 2 (bem-estar) manteve a coerência com o domínio temático original de bem-estar social e emocional^10^, apresentando uma boa consistência interna. Essa coerência é, também, consistente com os achados da versão malaica^15^.

Já o fator 3, que engloba apenas dois itens, conceitualmente distintos (função visual e sintomas visuais)^10^, contou com o agrupamento por critérios estatísticos, e não necessariamente por coerência temática. A literatura aponta que fatores com apenas dois itens tendem a apresentar confiabilidade limitada e são particularmente sensíveis à baixa correlação entre si^24^. A fraca correlação entre esses itens compromete a coesão interna do fator e levanta dúvidas quanto à sua utilidade como dimensão independente. Essa dificuldade foi acentuada pela especificidade do conteúdo do Item 1 (visão ao dirigir). A elevada taxa de respostas omissas nesse item reflete a realidade demográfica da amostra (estudantes que ainda não dirigem), o que comprometeu a coesão desse domínio. Esse fenômeno sublinha que, embora o ICR-QV seja uma ferramenta robusta, a sua aplicação em populações muito jovens deve considerar que certos domínios da qualidade de vida dependem da experiência de vida e da autonomia do respondente.

Apesar da fragilidade estatística do terceiro fator, optou-se pela manutenção da totalidade dos itens nessa fase de adaptação do questionário. Essa decisão fundamenta-se na preservação do valor teórico do construto original e na necessidade de garantir a comparabilidade com estudos internacionais que utilizam o mesmo referencial. Assim, considera-se que a versão portuguesa do QIRC, para avaliação do impacto do erro refrativo na qualidade de vida (ICR-QV), pode ser utilizada com segurança para avaliar o impacto da correção refrativa na qualidade de vida de jovens adultos, sobretudo nas dimensões de dificuldades/preocupações e bem-estar emocional.

Este estudo apresenta limitações. Primeiro, utilizou-se uma amostra por conveniência, composta exclusivamente por estudantes, o que limita a generalização dos resultados para outras faixas etárias e contextos sociodemográficos. A validade externa do instrumento deverá ser avaliada em estudos futuros com amostras mais heterogêneas. Segundo, este trabalho centrou-se na adaptação transcultural e na avaliação psicométrica inicial, não tendo explorado a sensibilidade do instrumento para distinguir grupos específicos, como míopes e hipermetropes. Terceiro, foi realizada apenas AFE, sem Análise Fatorial Exploratória (AFC) ou índices globais de ajuste do modelo. Assim, estudos futuros deverão confirmar a estrutura dimensional identificada em amostras independentes e avaliar a estabilidade da solução fatorial em diferentes populações.

A versão portuguesa do ICR-QV apresentou resultados satisfatórios na avaliação psicométrica inicial, com boa consistência interna global e elevada estabilidade temporal. A análise fatorial exploratória identificou uma estrutura dimensional distinta da originalmente proposta, tanto no número quanto na composição dos fatores, pelo que estes achados devem ser interpretados com cautela. Assim, recomenda-se que estudos futuros avaliem a estrutura dimensional do instrumento por meio de AFC, em amostras independentes e mais heterogêneas. Ainda assim, os resultados obtidos indicam que o ICR-QV pode constituir uma ferramenta útil para avaliar o impacto da correção refrativa na qualidade de vida em populações jovens de língua portuguesa.

## Materiais Suplementares

Anexo A
